# Diffuse Myocardial Injury With Diabetes-Related Advanced Glycation End Product Accumulation in Dilated Cardiomyopathy: Pathological Insights From an Autopsy Case

**DOI:** 10.7759/cureus.101298

**Published:** 2026-01-11

**Authors:** Sohei Kitazawa, Riko Kitazawa

**Affiliations:** 1 Molecular Pathology, Ehime University Graduate School of Medicine, Toon, JPN; 2 Diagnostic Pathology, Ehime University Hospital, Toon, JPN

**Keywords:** advanced glycation end products, cardiomyopathy, complication, cross-linking, diabetes

## Abstract

Diabetes mellitus is a systemic metabolic disorder associated with diverse long-term complications, among which cardiovascular involvement is a major cause of morbidity and mortality. A central pathogenic mechanism linking diabetes to tissue injury is the formation and accumulation of advanced glycation end products (AGEs), which exert deleterious effects through receptor-mediated signaling via the receptor for AGEs (RAGE) and receptor-independent collagen cross-linking. However, pathological evidence directly demonstrating the contribution of AGE accumulation to advanced cardiac dysfunction in human autopsy cases remains limited. We report an autopsy case of a Japanese man in his late 60s with long-standing idiopathic dilated cardiomyopathy who subsequently developed diabetes mellitus and progressive heart failure. His clinical course was complicated by secondary pulmonary hypertension, followed by hepatic and renal dysfunction, and ultimately resulted in death due to multiple organ failure. Gross examination revealed marked cardiac enlargement with severe biventricular dilatation without ventricular wall hypertrophy. Histologically, the myocardium showed diffuse interstitial and perimyocytic fibrosis with transmural myocardial atrophy. The pulmonary arteries demonstrated fibrous intimal thickening consistent with secondary pulmonary hypertension. Renal pathology showed arteriolosclerotic nephropathy with superimposed diabetic nephropathy, and the liver exhibited features of congestive hepatopathy. Immunohistochemical analyses demonstrated prominent myocardial deposition of pentosidine, a representative AGE, along with increased expression of the oxidative stress marker 8-hydroxy-2′-deoxyguanosine (8-OHdG) and immunoreactivity for RAGE. These findings suggest that diabetes-associated AGE accumulation and oxidative stress contributed to qualitative alterations of myocardial collagen within pre-existing diffuse fibrosis, potentially accelerating myocardial stiffening and progression of dilated cardiomyopathy.

## Introduction

Diabetes mellitus is a chronic metabolic disorder affecting multiple organs and is associated with a wide range of long-term complications that substantially contribute to morbidity and mortality [1-3]. Diabetic complications include both microvascular and macrovascular disorders, such as nephropathy, retinopathy, neuropathy, and cardiovascular disease [3]. In addition to these well-established entities, diabetes has been increasingly recognized to exert direct effects on the myocardium and vasculature, leading to structural remodeling and functional impairment that cannot be fully explained by ischemic injury or hypertension alone [4]. Such diabetes-related cardiac involvement represents an important contributor to the development and progression of heart failure [5].

A central pathogenic mechanism underlying these diverse diabetic complications is the formation and accumulation of advanced glycation end products (AGEs) [6]. AGEs are generated through non-enzymatic glycation reactions under conditions of chronic hyperglycemia and preferentially accumulate in tissues with low protein turnover, particularly within the extracellular matrix [6]. The pathological effects of AGEs are mediated through two principal mechanisms: receptor-mediated signaling via the receptor for advanced glycation end products (RAGE), which promotes oxidative stress and inflammation [7], and receptor-independent cross-linking of structural proteins such as collagen, resulting in increased tissue stiffness and impaired compliance [8]. Together, these mechanisms provide a unifying framework for diabetes-associated tissue injury, particularly within the cardiovascular system.

Although experimental studies have highlighted the importance of these mechanisms [9], pathological evidence directly linking AGE accumulation and collagen modification to advanced cardiac dysfunction in human autopsy cases remains limited. The present case provides morphological insight into the potential contribution of diabetes-associated AGEs to the progression of dilated cardiomyopathy and secondary pulmonary hypertension.

## Case presentation

A Japanese man in his late 60s presented with a nocturnal cough and progressive dyspnea and was referred to our hospital. He had been diagnosed with idiopathic dilated cardiomyopathy 13 years earlier and subsequently developed chronic heart failure. Three years after the initial diagnosis, he was diagnosed with diabetes mellitus. Although detailed longitudinal data regarding glycemic control, including serial HbA1c measurements, were not consistently available because of the retrospective autopsy-based nature of this case and incomplete access to long-term outpatient records, the patient was clinically considered to have persistently poor glycemic control during the course of his illness, based on the medical history and overall clinical context. In the year preceding his death, he was treated for severe sleep apnea syndrome; however, home oxygen therapy failed to provide clinical benefit. His clinical condition progressively deteriorated, and he ultimately developed severe hepatic and renal dysfunction associated with low cardiac output syndrome and died of multiple organ failure.

Autopsy findings

Macroscopic Findings

**Figure 1:**
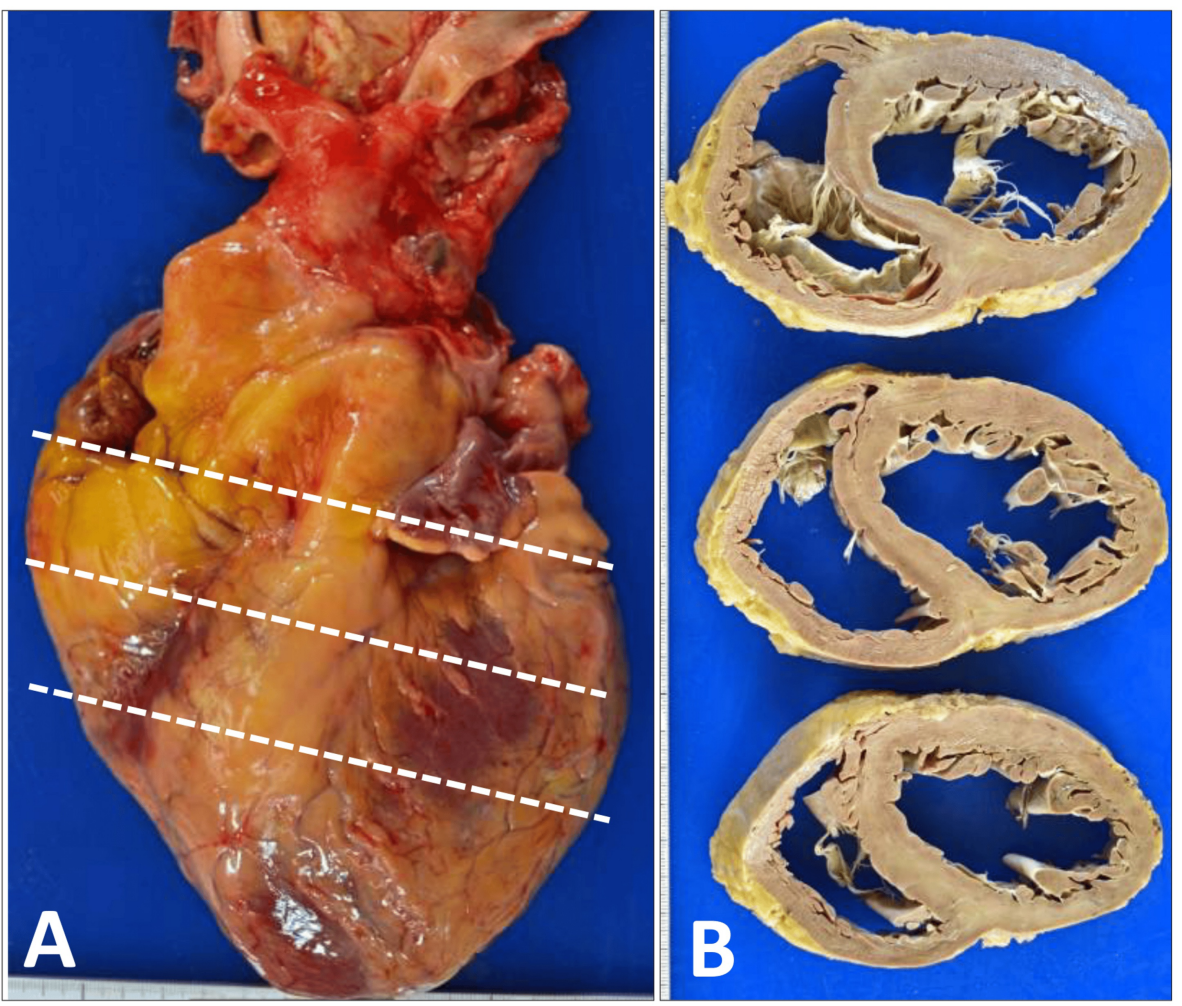
Gross appearance of the heart at autopsy. (A) External view of the heart obtained at autopsy. The heart weighed 646 g and was markedly enlarged, exhibiting an elongated oval configuration. (B) Serial transverse sections of the heart after fixation, cut along the three white dashed lines indicated in panel (A) and arranged from top to bottom. The lower part of the figure corresponds to the anterior aspect of the heart; the left ventricle is located on the right side of the image, and the right ventricle on the left. The heart is globally enlarged, and the right ventricular wall shows mild thickening, consistent with pulmonary hypertension. No focal lesions are identified macroscopically, and the myocardial walls of both ventricles appear uniform.

An autopsy was initiated two hours after death. The heart weighed 646 g and showed marked dilatation of both ventricular cavities (Figure 1A) without significant thickening of the ventricular walls on cut section (Figure 1B); no myocardial infarction was identified, and the coronary arteries exhibited atherosclerotic changes. The lungs weighed 368 g on the left and 503 g on the right, with pale yellow, clear pleural effusions measuring approximately 200 mL on the left and a small amount on the right; mild adhesions between the upper and lower lobes of the left lung were present and were easily separated manually. The liver weighed 1,098 g and demonstrated a mottled appearance on the cut surface, consistent with congestive hepatopathy. The kidneys weighed 193 g on the left and 196 g on the right, and showed irregular surfaces with cyst formation.

Microscopic Findings

**Figure 2:**
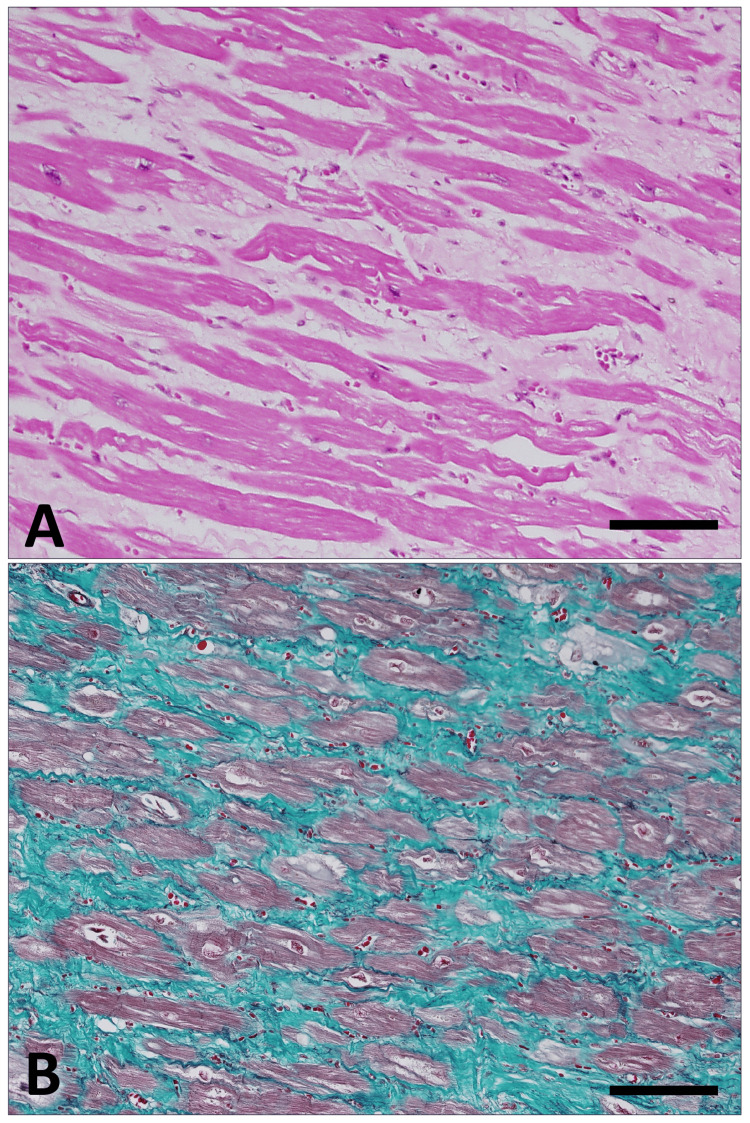
Histological findings of the myocardium. (A) Hematoxylin and eosin staining shows pale eosinophilic fibrotic areas expanding between intensely eosinophilic myocardial fibers. (B) Elastica–Masson staining highlights collagen fibers, which are stained green by the Masson component, more clearly delineating the extent of interstitial fibrosis. Scale bars represent 150 μm.

In the left ventricular wall and interventricular septum, transmural myocardial atrophy, perimyocytic fibrosis, and diffuse interstitial fibrosis were observed (Figure 2A-2B). In the right ventricular wall, fibrous proliferation was noted predominantly in perivascular areas. The coronary arteries, including the left anterior descending artery, left circumflex artery, and right coronary artery, showed fibrous intimal thickening accompanied by calcification and inflammatory cell infiltration, resulting in luminal stenosis of up to approximately 50%. 

In both lungs, fibrous intimal thickening of the pulmonary arterial walls was observed from the hilar to peripheral regions, consistent with secondary pulmonary hypertension. Alveolar spaces contained hemosiderin-laden macrophages (heart failure cells). Mild inflammatory cell infiltration was present around the bronchi; however, no histological evidence suggestive of pneumonia was identified. In the liver, mild pericentral fibrosis, hemorrhage, hepatocellular necrosis, and dropout were observed. There was no portal expansion or inflammatory cell infiltration, and no findings suggestive of chronic hepatitis. The spleen showed marked congestion, with red blood cells filling the splenic sinusoids, consistent with congestive splenomegaly. In both kidneys, wedge-shaped cortical lesions were noted, characterized by glomerulosclerosis, thickening of the tubular basement membranes, and interstitial fibrous proliferation, consistent with arteriolosclerotic nephropathy. In addition, nodular lesions within the glomeruli were observed, indicating superimposed diabetic nephropathy. The prostate demonstrated cystically dilated glands with a serrated architecture, consistent with benign prostatic hyperplasia. The aorta showed fibrous intimal thickening with atheroma formation composed of cholesterol crystal deposition and inflammatory cell infiltration. The bone marrow exhibited hypercellularity of approximately 70%, with preservation of all three hematopoietic lineages. No additional complications, such as infectious diseases that could have precipitated an acute deterioration of heart failure, were identified.

**Figure 3:**
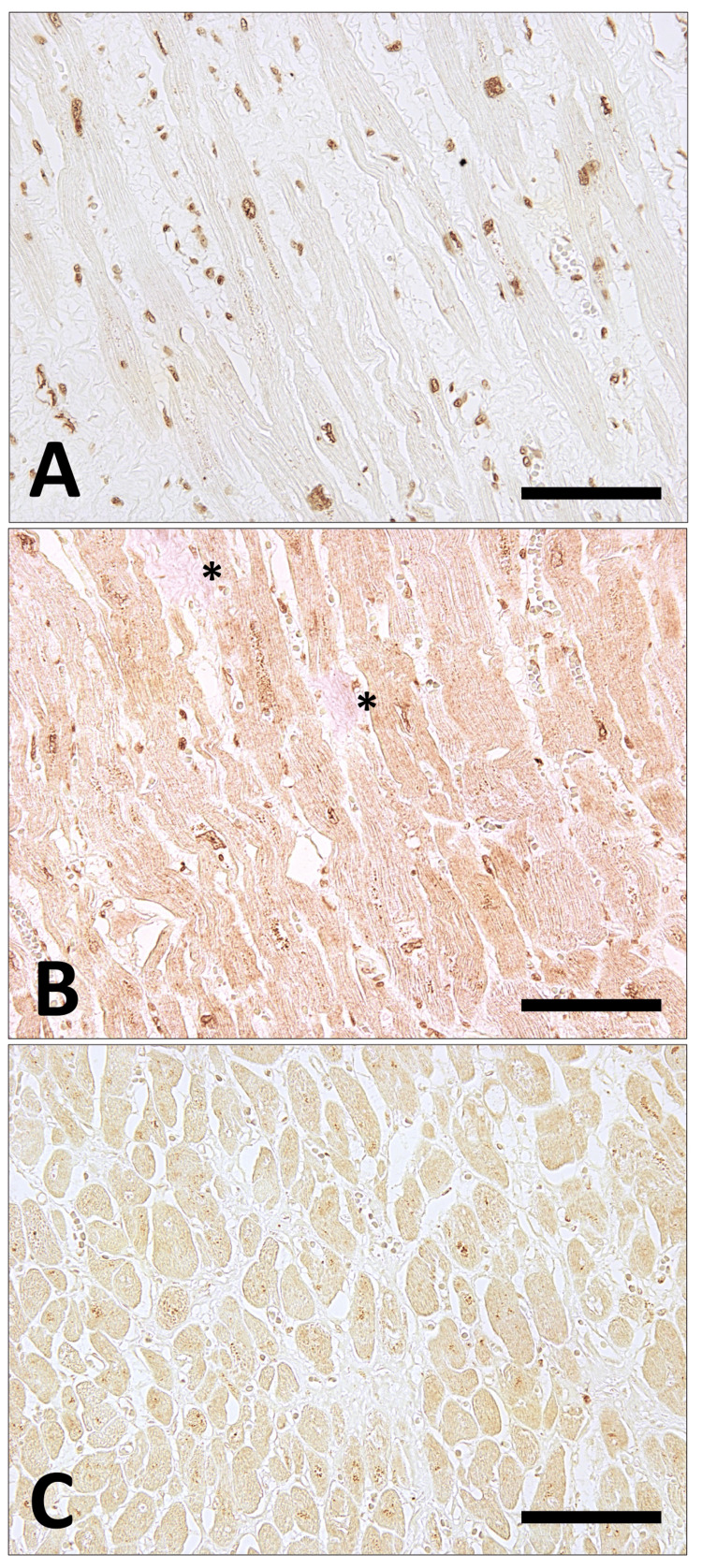
Immunohistochemical localization of oxidative stress marker 8-OHdG, AGE (pentosidine), and RAGE in the myocardium. (A) Immunostaining for 8-OHdG shows positive nuclear staining in almost all cardiomyocytes. Nuclear positivity is also observed in a subset of vascular endothelial cells. (B) Pentosidine immunostaining demonstrates widespread positivity in the perinuclear regions and cytoplasm of cardiomyocytes, as well as in areas of interstitial fibrosis (asterisk). (C) Immunoreactivity for RAGE is relatively weak but is diffusely detected in cardiomyocytes. Scale bars represent 150 μm. AGE: advanced glycation end products; RAGE: receptor for advanced glycation end products

Immunohistochemical analysis demonstrated positive staining for 8-hydroxy-2′-deoxyguanosine (8-OHdG), an oxidative stress marker, using a monoclonal anti-8-OHdG antibody (clone N45.1, Japan Institute for the Control of Aging, Shizuoka, Japan) (Figure 3A) and for pentosidine, a representative AGE, using a monoclonal anti-pentosidine antibody (KH012-02, TransGenic Inc., Kumamoto, Japan) (Figure 3B) within the myocardium, which suggests a contributory role of hyperglycemia in myocardial injury. In addition, immunoreactivity for the receptor for AGEs (RAGE) was detected in the myocardium using a polyclonal anti-RAGE antibody (sc-365154, Santa Cruz Biotechnology, Inc., CA) (Figure 3C).

## Discussion

This case represents a fatal course in which dilated cardiomyopathy progressed to advanced heart failure, followed by respiratory failure due to secondary pulmonary hypertension, and ultimately culminated in multiple organ failure as a result of decompensated circulatory insufficiency. Dilated cardiomyopathy is pathologically characterized by diffuse myocardial fibrosis, which contributes to progressive ventricular remodeling and impaired cardiac function [10,11]. In the present case, extensive interstitial and perimyocytic fibrosis was observed throughout the myocardium, providing a structural substrate for chronic systolic dysfunction.

Notably, immunohistochemical analyses demonstrated the deposition of pentosidine within the myocardium and surrounding fibrotic areas (Figure 3B). These findings indicate a spatial association between myocardial fibrosis and diabetes-related AGE accumulation. However, given the descriptive nature of this single autopsy case and the absence of quantitative assessment, the observations should be interpreted as morphological correlations rather than evidence of a causal relationship. Accordingly, the present findings are best viewed as hypothesis-generating, suggesting that diabetes-associated glycation may influence the qualitative properties of collagen within fibrotic myocardium in dilated cardiomyopathy.

In diabetes mellitus, non-enzymatic glycation of collagen leads to the formation of abnormal intermolecular cross-links, resulting in increased stiffness, reduced elasticity, and impaired degradation of collagen fibers [12,13]. Such qualitative alterations have been well documented in diabetic bone tissue, where excessive collagen cross-linking renders the matrix brittle despite preserved or even increased collagen content [14]. By analogy, a similar mechanism may plausibly operate in the myocardium, where AGE-mediated collagen modification within pre-existing fibrotic areas could increase myocardial stiffness. Nevertheless, in the absence of comparative control tissues, such as non-diabetic dilated cardiomyopathy or age-matched normal myocardium, the specificity of this finding for diabetes-related myocardial remodeling cannot be determined. Future studies incorporating appropriate control cohorts and quantitative analyses will be required to clarify this issue.

Furthermore, comparable pathological changes were identified in the pulmonary vasculature in the present case. The pulmonary arterial walls showed fibrous intimal thickening, consistent with secondary pulmonary hypertension. It is conceivable that AGE accumulation and collagen cross-linking also affected the extracellular matrix of the pulmonary arterial wall, increasing vascular stiffness and resistance [15]. As with the myocardial findings, this interpretation remains speculative, as direct quantification of AGE burden and comparative vascular controls were not available in the present study. Nonetheless, the coexistence of myocardial and pulmonary vascular fibrosis raises the possibility that systemic metabolic stress associated with diabetes may contribute to multiorgan extracellular matrix remodeling in advanced heart failure.

In addition to metabolic factors, intercurrent systemic insults may further modify disease progression in patients with advanced cardiomyopathy. Recent evidence indicates that SARS-CoV-2 infection is associated with sustained myocardial injury, chronic inflammation, and endothelial dysfunction, particularly in patients with pre-existing heart failure. A recent systematic review demonstrated that COVID-19 confers a long-term cardiovascular burden, including increased risks of functional decline, rehospitalization, and mortality in this population [16]. Although a direct contribution of intercurrent infection cannot be established in the present case, such inflammatory and endothelial perturbations may have acted as compounding factors in accelerating cardiopulmonary deterioration.

Together, these observations support a conceptual framework in which diabetes-associated accumulation of AGEs, particularly pentosidine, is associated with qualitative collagen modification within diffusely fibrotic myocardium and pulmonary vasculature. Rather than establishing causality, the present autopsy findings provide morphological evidence consistent with a potential interaction between metabolic stress, extracellular matrix remodeling, and advanced cardiac dysfunction. Although causality cannot be conclusively established from a single autopsy case, the present findings highlight a plausible pathological link between diabetic collagen modification and the rapid progression from compensated dilated cardiomyopathy to decompensated heart failure with secondary pulmonary hypertension. Validation of this hypothesis will require larger autopsy series, comparative analyses, and integrative clinical-experimental studies.

## Conclusions

This autopsy case suggests that diabetes-associated accumulation of AGE products, particularly pentosidine, may contribute to myocardial injury and functional deterioration in the setting of diffusely fibrotic myocardium in dilated cardiomyopathy. The present findings provide morphological evidence supporting a potential role of diabetes-related metabolic stress in accelerating the pathological progression of dilated cardiomyopathy.
